# Feasibility of a refurbished shipping container as a transportable laboratory for rapid SARS-CoV-2 diagnostics

**DOI:** 10.1099/acmi.0.000346

**Published:** 2022-04-19

**Authors:** Stephen Muhi, Nick Tayler, Tuyet Hoang, Jacqueline Prestedge, Jean Y. H. Lee, Susan A. Ballard, Nicole Isles, Andrew Wlodek, Arran Greenhalgh, Deborah A. Williamson, Benjamin P. Howden, Timothy P. Stinear

**Affiliations:** ^1^​ Microbiological Diagnostic Unit Public Health Laboratory, The University of Melbourne at the Peter Doherty Institute for Infection and Immunity, Melbourne, Australia; ^2^​ Victorian Infectious Diseases Service, Royal Melbourne Hospital, Melbourne, Australia; ^3^​ Department of Microbiology and Immunology, The University of Melbourne at the Peter Doherty Institute for Infection and Immunity, Melbourne, Australia; ^4^​ Department of Emergency Medicine, Royal Melbourne Hospital, Melbourne, Australia; ^5^​ Department of Infectious Diseases, Monash Health, Clayton, Victoria, Australia; ^6^​ Business Services, University of Melbourne, Melbourne, Victoria, Australia; ^7^​ Geneworks Molecular and Cell Biology, 28 Dalgleish St, Thebarton, 5031, Australia; ^8^​ Department of Microbiology, Royal Melbourne Hospital, Melbourne, Australia

**Keywords:** COVID-19, SARS-CoV-2, point-of-care, rapid, antigen

## Abstract

**Background:**

Australia’s response to the coronavirus disease 2019 (COVID-19) pandemic relies on widespread availability of rapid, accurate testing and reporting of results to facilitate contact tracing. The extensive geographical area of Australia presents a logistical challenge, with many of the population located distant from a laboratory capable of robust severe acute respiratory syndrome coronavirus 2 (SARS-CoV-2) detection. A strategy to address this is the deployment of a mobile facility utilizing novel diagnostic platforms. This study aimed to evaluate the feasibility of a fully contained transportable SARS-CoV-2 testing laboratory using a range of rapid point-of-care tests.

**Method:**

A 20 ft (6.1 m) shipping container was refurbished (GeneWorks, Adelaide, South Australia) with climate controls, laboratory benches, hand-wash station and a class II biosafety cabinet. Portable marquees situated adjacent to the container served as stations for registration, sample acquisition and personal protective equipment for staff. Specimens were collected and tested on-site utilizing either the Abbott ID NOW or Abbott Panbio rapid tests. SARS-CoV-2 positive results from the rapid platforms or any participants reporting symptoms consistent with COVID-19 were tested on-site by GeneXpert Xpress RT-PCR. All samples were tested in parallel with a standard-of-care RT-PCR test (Panther Fusion SARS-CoV-2 assay) performed at the public health reference laboratory. In-laboratory environmental conditions and data management-related factors were also recorded.

**Results:**

Over a 3 week period, 415 participants were recruited for point-of-care SARS-CoV-2 testing. From time of enrolment, the median result turnaround time was 26 min for the Abbott ID NOW, 32 min for the Abbott Panbio and 75 min for the Xpert Xpress. The environmental conditions of the refurbished shipping container were found to be suitable for all platforms tested, although humidity may have produced condensation within the container. Available software enabled turnaround times to be recorded, although technical malfunction resulted in incomplete data capture.

**Conclusion:**

Transportable container laboratories can enable rapid COVID-19 results at the point of care and may be useful during outbreak settings, particularly in environments that are physically distant from centralized laboratories. They may also be appropriate in resource-limited settings. The results of this pilot study confirm feasibility, although larger trials to validate individual rapid point-of-care testing platforms in this environment are required.

## Introduction

Coronavirus disease 2019 (COVID-19), caused by severe acute respiratory syndrome coronavirus 2 (SARS-CoV-2), manifests with varying severity in humans, from asymptomatic disease or influenza-like illness through to severe pneumonia and death [1]. SARS-CoV-2 is a β-coronavirus of the subgenus *Sarbecovirus* and the subfamily *Orthocoronavirinae*. It is an enveloped non-segmented positive-sense RNA virus [2–4].

To date, testing for SARS-CoV-2 in Australia has mostly been performed in centralized laboratories. Mobile laboratories in shipping containers have been manufactured in Australia and are now available for deployment [5, 6] and similar shipping container laboratories have been deployed elsewhere [7]. However, to our knowledge, portable physical containment level 2 (PC2)-capable laboratories have not been used for on-site COVID-19 testing in the Australian context.

The current standard-of-care diagnostic test for SARS-CoV-2 is laboratory-based molecular detection of viral RNA in human clinical specimens, predominantly using reverse transcriptase polymerase chain reaction (RT-PCR) [4, 8]. The World Health Organization (WHO) recommends an assay that detects at least two independent targets of the viral genome and recommends interpreting the results of commercial assays in accordance with their instructions for use [9]. These tests have excellent sensitivity and specificity, but one limitation is a relatively long time to result, which may delay contact tracing. Novel techniques have been developed for rapid detection of SARS-CoV-2, including the Abbott ID NOW (Abbott, Chicago, USA) that aims to provide a result within 13 min of sample insertion [10].

Another method for detection is using a point-of-care test (POCT) to detect the SARS-CoV-2 nucleocapsid protein using monoclonal antibodies, combined with colloidal gold nanoparticles, to give a visual representation of whether the antigen is present on a membrane using a lateral flow immunoassay [3, 11]. This technique has been used for the detection of SARS-CoV-2 [12–14] and the Abbott Panbio COVID-19 Ag Rapid Test Device (Abbott, Chicago, USA) is an example of this technology approved for supply by the Australian Therapeutic Goods Administration.

Our hypothesis was that a self-contained portable laboratory, combined with on-site sample acquisition, is a feasible method for testing for COVID-19 and may reduce turnaround time to results.

## Methods

The study was performed between 15 November and 5 December 2020. At this time there were no known active COVID-19 cases in the Melbourne area. The testing site was placed on the grounds of the University of Melbourne, in a car park that was chosen due to its proximity to the surrounding residential college dormitories.

### Site design

**Fig. 1.**
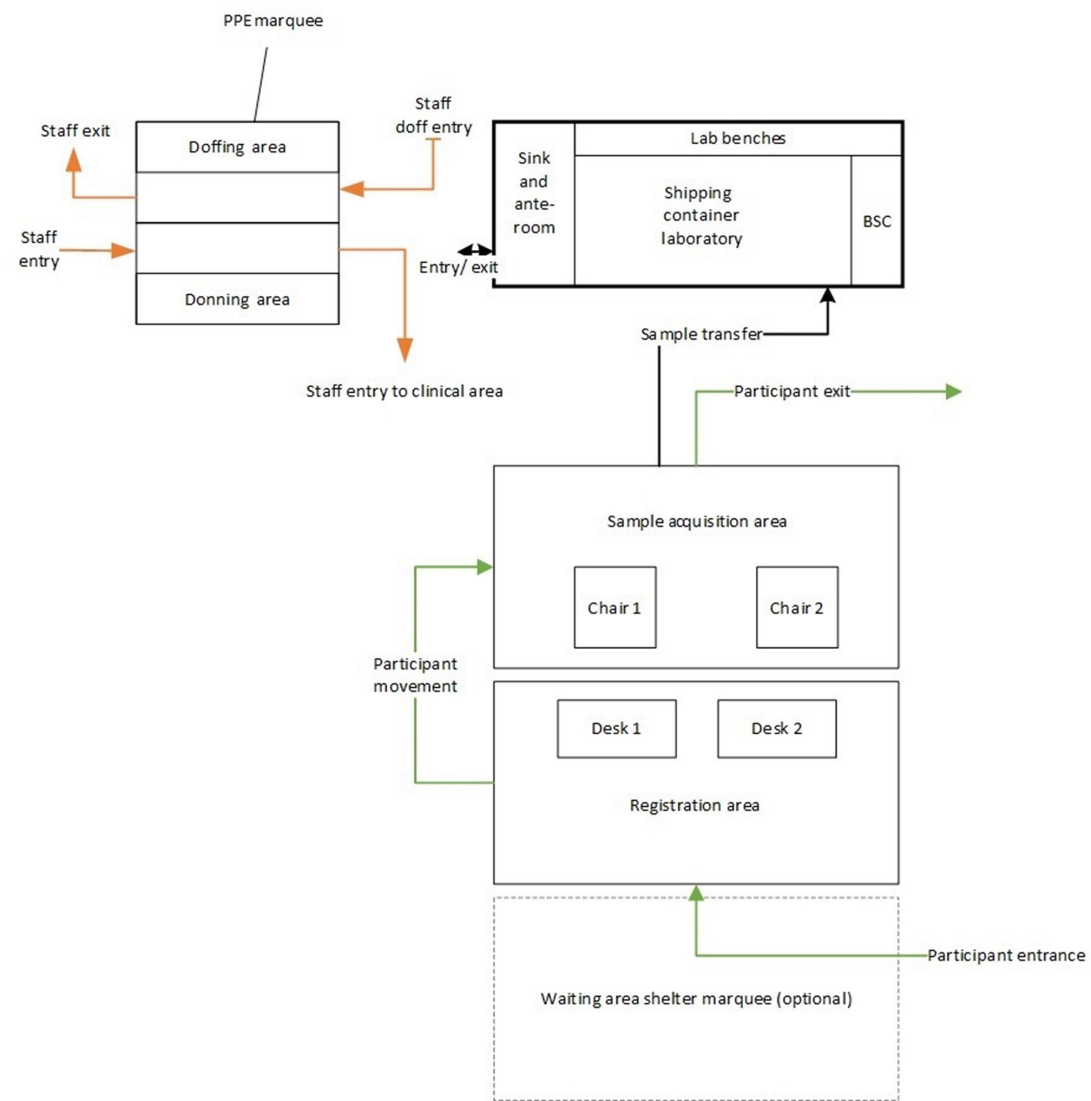
An overview of the portable laboratory and testing site showing the registration area, sample acquisition area and flow of persons and samples. BSC, biosafety cabinet.

The testing site consisted of two 6×3 m adjacent marquee tents with removable sidewalls, one for participant registration and one for sample acquisition. An additional 6×3 m marquee was available for shelter for waiting participants and was used as required. A separate 3×3 m marquee was used exclusively for the donning and doffing of personal protective equipment to maintain staff safety (donning and doffing sections were separated by internal bollards) (Fig. 1). Waste was removed by specialist waste contractors daily.

**Fig. 2.**
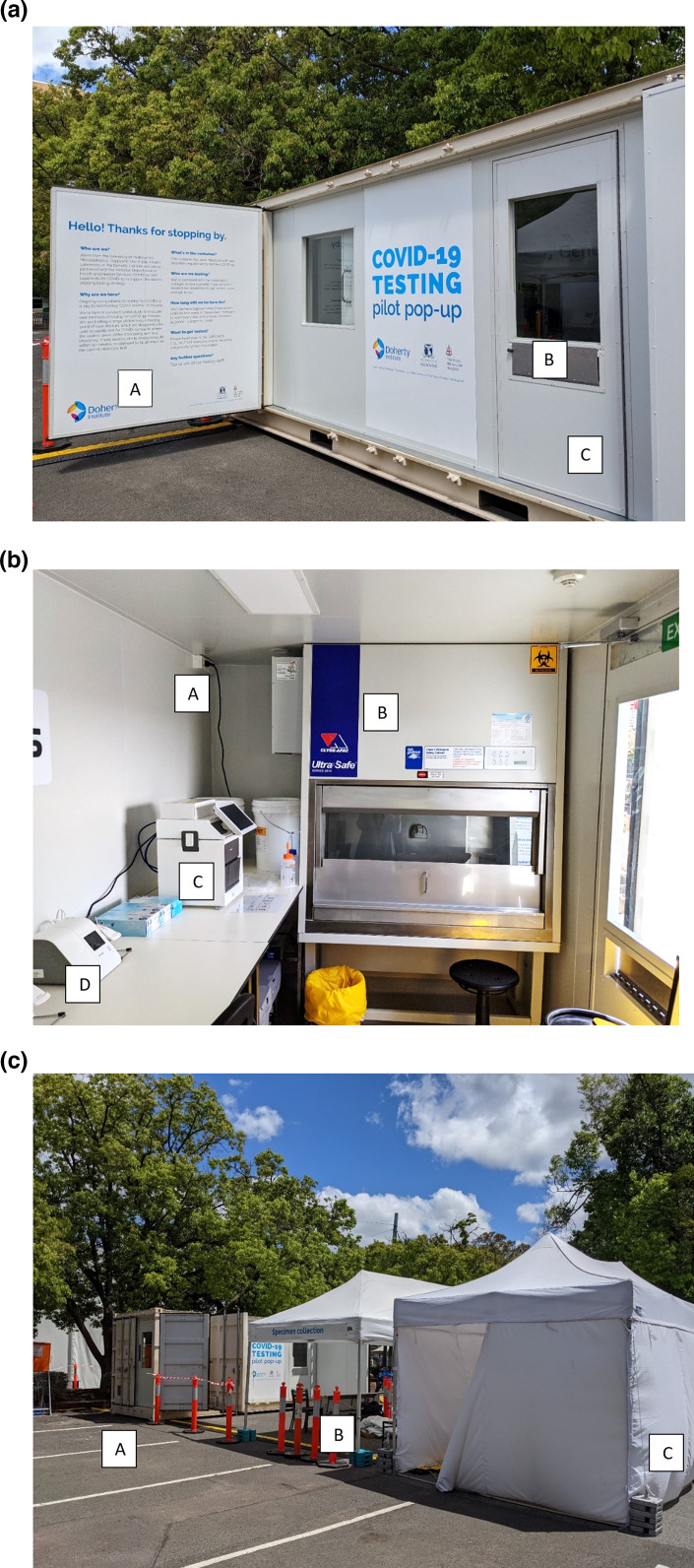
(a) An external view of the laboratory. A: information for participants. B: specimen reception articulated flap. C: emergency exit door. (b) internal view of the laboratory. A: integrated mains electricity. B: Clyde-Apac Ultra Safe Class II biosafety cabinet. C: GeneXpert Xpress system. D: Abbott ID NOW instrument. (c) external view of laboratory and sample acquisition area. A: main entrance and exit for laboratory. B: sample acquisition area. C: test registration marquee.

A standard steel 20 ft (6.1 m) shipping container was refurbished (GeneWorks, Adelaide, South Australia) to meet specifications for a PC2 laboratory (Fig. 2a–c). In brief, a side-opening shipping container was fitted with internal doors, climate control, a biosafety cabinet and ventilation systems in order to meet PC2 criteria. An articulated flap allowed samples to be safely transferred inside the container.

Within the shipping container, chairs and stainless steel laboratory benches were installed alongside a Clyde-Apac UltraSafe Class II biological safety cabinet (AES Environmental, NSW, Australia) with National Association of Testing Authorities (NATA) certification when on site. Electricity supply was from dual 10 A power inputs with dual uninterruptable power supplies and backup power of approximately 3000 V⋅A each.

A sink with an automatic sensor faucet was fitted with wastewater connection and inlet water with backwash protection. The floors were composed of laboratory linoleum with raised edges and sealed wall gaps. Safety features included a dual exit system with emergency lighting. Climate control was provided by Hitachi systems split system reverse cycle air conditioning and temperature was monitored using Tinytag View 2 TV-4505 (Gemini Data Loggers, Chichester, UK), whilst mobile internet connectivity was managed by an industrial G800V2 4G router (USR IOT, Shandong, PR China).

### Sample acquisition

Participant registration was completed using mobile internet-enabled tablet computers (Apple iPad seventh generation, software version 14.2) with a bluetooth-enabled barcode scanner (POS-mate, Adelaide, Australia). Participants were allocated a Victorian Department of Health and Human Services (DHHS) tracking number for the standard-of-care test (SOCT) result, as well as a de-identified study identification number.

Nursing staff collected a combined bilateral throat and single nostril deep nasal swab for the SOCT, which was immediately placed in 3 ml of universal transport medium (UTM). This was followed by a swab in the contralateral nostril for novel testing platforms, utilizing the swab provided with the novel test device. Samples were then immediately transferred to the transportable PC2 laboratory via the flap, which was adjacent to the sample collection area. SOCT were transferred twice per day to the Microbiological Diagnostic Unit Public Health Laboratory (MDU PHL) for RT-PCR utilizing the Aptima SARS-CoV-2 assay (Hologic, Marlborough, MA, USA).

### Data management

The portable laboratory setup required innovative data management solutions to deliver a SOCT result to the participant as well as a POCT to the study team. All staff received training in the use of data management software to ensure that results were accurately recorded. SOCTs were registered using the DHHS Test Tracker electronic COVID-19 test registry [15] with a unique identifier allocated to each sample, called a d-number, which was available in the form of a quick response (QR) code, which provided an internet link to the registration page for that specific sample. POCTs were tracked separately using an alphanumeric sample identifier and a secure REDCap database [16]. Participants were not informed of POCT results.

Prelabelled sample packs were prepared and assigned to the participant on arrival with the SOCT d-number forming the core identifier. Although mobile internet-enabled devices were used for participant registration with an encrypted connection to both the DHHS Test Tracker system and the REDCap database, a paper record of all participants was also maintained as a backup.

Time-stamped electronic data capture enabled testing turnaround times to be accurately recorded. On-site devices with electronic data outputs (the Abbott ID NOW and Xpert Xpress SARS-CoV-2) had anonymous data exported into a comma separated values (.csv) file on a USB key and uploaded into the REDCap database under the sample identifier, which was the same across all test platforms. The Abbott Panbio result was interpreted by two technicians independently and entered into the REDCap database. In addition, a high-resolution photograph of the antigen test result and sample identifier was uploaded using the camera function of the internet-enabled tablet computer (Apple iPad seventh generation). To standardize digital photography, the tablet computer was placed on a box stand 4 cm directly above the bench with an attached macro lens (Black Eye 3-in-1) with a generic ring light.

Electronic test request messages for the SOCT were generated and managed using Test Tracker and imported into the MDU PHL laboratory information system (LIS). The results of the SOCT were imported into LIS and negative results were reported to participants via short message service (SMS) and positive results to the assigned clinician.

### Recruitment

Participants were recruited from the University of Melbourne; both staff and students were invited to attend. Recruitment was supported by a range of communication strategies, including engagement with the leadership of the surrounding college dormitories, University of Melbourne intranet and email promotion, as well as signage and posters adjacent to the site inviting participants who were nearby.

## Results

A total of 415 participants presented for COVID-19 testing at the university site. The median age was 26 (range 18–86) and there were 206 females, 208 males and 1 unspecified. Twenty-four participants reported symptoms consistent with COVID-19. Overall, the portable testing site provided a suitable environment for sample collection and processing of samples, regardless of the platform used. The ID NOW showed similar performance characteristics to other studies, with 1 false positive reported from 286 valid tests, giving a negative performance agreement (NPA) of 99.6 %. There were five invalid tests results, which represented 1.7 % of all tests performed.

The Abbott Panbio Ag test was utilized in the portable laboratory setting, and the climate control of the container laboratory ensured that the test devices stayed within the manufacturer-specified temperature range. It showed similar performance characteristics to other studies, with 100 % NPA and returned no invalid results. The Xpert Xpress SARS-CoV-2 test had 100 % NPA with the standard-of-care test from 25 tests completed. A single sample returned an invalid result from the mobile laboratory device, which was repeated and found to be negative (4 % invalid).

Temperature data showed a mean internal temperature of the laboratory of 19.5 °C, with a range of 14.3–26.0 °C (Fig. S1, available in the online version of this article). The external temperature ranged from 10.6–36.0 °C during the study period [17, 18]. If outside temperatures were greater than 30 °C, the test site was closed to ensure the health and safety of staff working outdoors; this occurred twice during the study period. Due to a malfunction of the data logger, humidity tracking data were not available.

### Time to result

The turnaround times of novel testing platforms were calculated from initial registration to reporting, as summarized in Table 1. Due to technical malfunction of the REDCap database, 146 of 291 results were missing registration timestamps for the Abbott ID NOW device. The data export from the Xpert Xpress did not record an accurate time of test for four samples. Formal RT-PCR results were reported to participants within 24 h of test registration via SMS, as is standard practice in Victoria.

**Table 1.** Median time to results reporting for each device assessed in the portable laboratory

Device	Median time (min)	Minimum time (min)	Maximum time (min)	No. with valid timestamps	
Abbott Panbio	32	22	127	113	All captured by REDCap
Abbott ID NOW	26	16	93	145	REDCap redesigned to capture timestamp after 141 results
Xpert Xpress SARS-CoV-2	75	63	94	21	Four missing time data from Xpert Xpress data output

## Discussion

Overall, the portable PC2 laboratory provided a controlled environment for the safe processing of SARS-CoV-2 tests and is a valuable proof of concept for decentralized testing for other communicable diseases where proximity of the analytical capability provides access to timely results, particularly in remote locations. The range of SARS-CoV-2 testing platforms that were utilized in the portable laboratory demonstrated similar performance characteristics to studies performed in a hospital setting and traditional laboratory environments. This may enable rapid testing using novel platforms in settings where access is otherwise restricted. Specifically, the PC2 capable laboratory with a biosafety cabinet allowed on-site RT-PCR testing to be performed using UTM specimens.

The climate in Melbourne, Australia, during late spring and early summer generally allowed for specimen collection to be performed in marquees with minimal staff discomfort. Whilst sample collection activities ceased if outdoor temperatures exceeded 30 °C, the laboratory climate control systems maintained a permissible operating temperature for equipment and staff. Sample collection and processing continued in heavy rainfall (30.2 mm/24 h), although environmental humidity of 100 % [17, 18] may have contributed to condensation on the inner surfaces of the laboratory and inside the lids of the Abbott ID NOW instruments. Maintaining climate control within the required specifications for each instrument may limit the deployment of our described facility to geographical areas with extreme temperatures.

A significant limitation of this study was the limited sample size of tests performed using each novel platform and the absence of COVID-19 in the study population. The results of this pilot study demonstrate feasibility but cannot draw meaningful conclusions about the validation of novel platforms in this environment, although during a period without local community transmission this was not the primary aim of the study.

The Xpert Xpress SARS-CoV-2 operated within a container laboratory and provided diagnostic testing results much more rapidly than transferring the samples to a laboratory for formal RT-PCR testing. However, the availability of the Xpert Xpress SARS-CoV-2 assay may limit widespread use [19, 20]. The Abbott ID NOW device performed with similar characteristics as within other settings [21], although the reduced sensitivity of this device limits its use as a rule-out test [22]. With a throughput of four samples per instrument per hour for the Xpert Xpress, and two to seven samples per instrument per hour for the Abbott ID NOW, these molecular-based tests have limited capacity for use as a high-throughput screening tool.

The Abbott Panbio antigen test, which has been comprehensively evaluated elsewhere [22], enables higher throughput at point of care, while also providing a liquid sample in a buffer solution that may be useful for other testing modalities, such as RT-PCR, subject to validation. Furthermore, this method does not require additional laboratory instrumentation. However, data management is manual, with no automated reading or LIS connection available, and thus subject to transcription errors. Given the current extremely low prevalence in Australia, a positive antigen test is more likely to represent a false positive, therefore urgent confirmatory testing is still required. A possible approach could be to perform high-throughput antigen testing at peripheral sites and only transfer presumptively positive samples to a nearby PC2-enabled container laboratory for reflex confirmatory testing. Further work is needed to establish whether antigen testing is effective in asymptomatic individuals in a low-prevalence setting.

Data management, which was complicated by performing a SOCT alongside research tests with different reporting requirements, was sufficient to provide accurate and traceable results from a field setting. This relied on the availability of mobile internet for test registration and a paper backup was maintained in the event that the internet became unavailable. Where cellular internet may not be available in geographically isolated regions, data could also be stored locally for later upload, if required.

## Conclusion

The transportable PC2 laboratory described here permits high-quality, decentralized, rapid COVID-19 diagnostics, and may be useful for other communicable diseases in outbreak settings. Temperature control conditions were suitable, although humidity may have contributed to condensation within the container laboratory. Data were captured using available software for the majority of samples, but not all were timestamped due to a technical malfunction. The results of this pilot study confirm feasibility, although larger trials to validate individual rapid point-of-care testing platforms in this environment are required.

## Supplementary Data

Supplementary material 1
