# Supplementary figures and images for: Feasibility of a refurbished shipping container as a transportable laboratory for rapid SARS-CoV-2 diagnostics
Source: Access Microbiol. 2022 Apr 19;4(4):000346. doi: 10.1099/acmi.0.000346 (PMC9260087; doi:10.1099/acmi.0.000346)

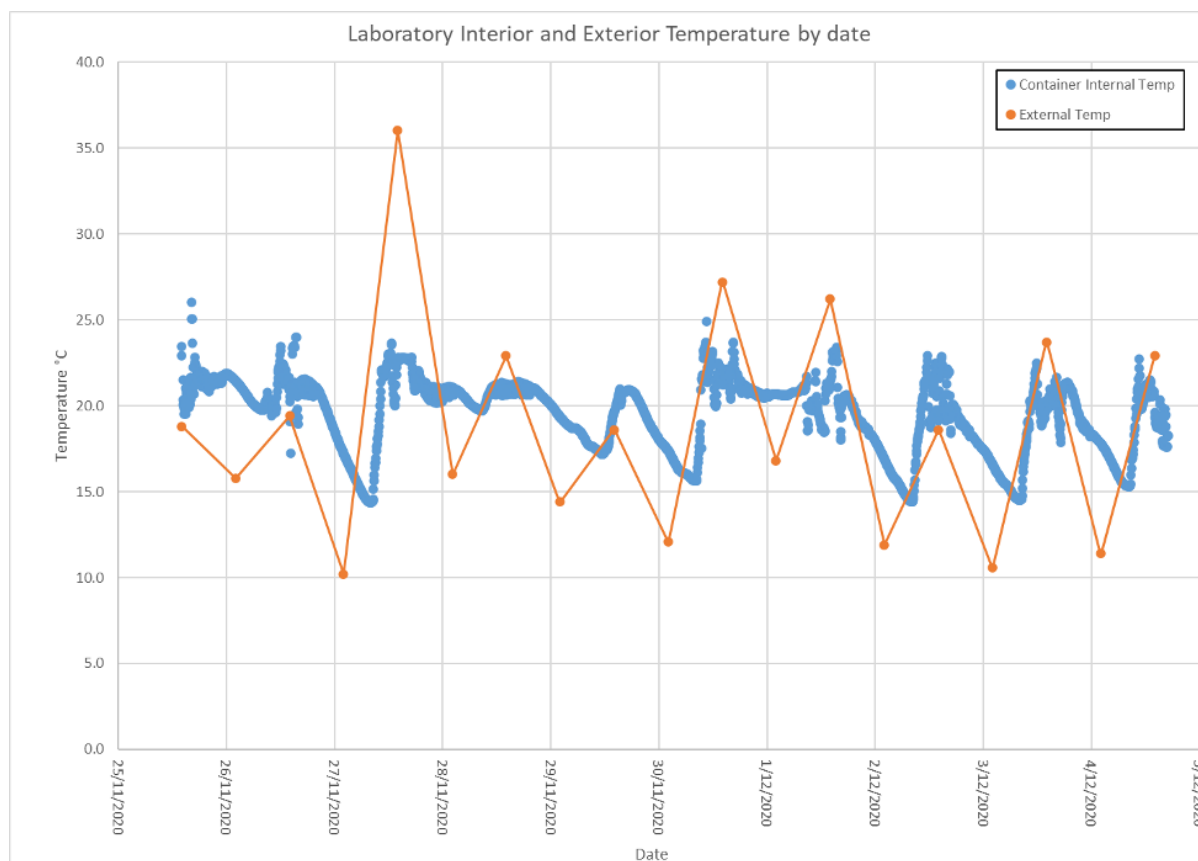

Supplementary Figure 1: Log of internal container temperature against time for study duration.

Supplement: Supplementary material 1 [file acmi-4-0346-s001.pdf]
